# Impact of Measured and Predicted Patient–Prosthesis Mismatch on Quality of Life Following Transcatheter Aortic Valve Implantation

**DOI:** 10.1016/j.shj.2025.100759

**Published:** 2025-11-10

**Authors:** Karim Al-Azizi, Mohamad Bader Abo Hajar, Taylor Pickering, Ghadi Moubarak, Cody W. Dorton, Kyle A. McCullough, Jonathan Ladner, Maya Elias, Colleen Parro, Shelby L. McCoy, Uzair Saeed, Tsung-Wei Ma, Sarah Hale, Swapnil Gupta, Katherine B. Harrington, Justin M. Schaffer, Asim Mohiuddin, William T. Brinkman, Amro Alsaid, Janaki Manne, Ralph Matar, Prajakta Phatak, Sibi Thomas, Zuyue Wang, Robert Stoler, Subhash Banerjee, Yashasvi Chugh, Timothy Mixon, Robert J. Widmer, Angel Caldera, Jose Condado Contreras, Anita Krueger, William Gray, Julius Ejiofor, Imran Baig, Srinivasa Potluri, J. Michael DiMaio, Molly Szerlip, Michael J. Mack

**Affiliations:** a Department of Cardiology, Baylor Scott & White The Heart Hospital Plano, Plano, Texas, USA; b Department of Research, Baylor Scott & White Research Institute, Dallas, Texas, USA; c Department of Cardiothoracic Surgery, Baylor Scott & White The Heart Hospital Plano, Plano, Texas, USA; d Department of Cardiology, Baylor University Medical Center, Dallas, Texas, USA; e Department of Cardiology, Baylor Scott & White Medical Center, Temple, Texas, USA; f Department of Cardiology, Baylor Scott & White Medical Center, Round Rock, Texas, USA; g Department of Cardiothoracic Surgery, Baylor Scott & White All Saints Medical Center, Fort Worth, Texas, USA; h Department of Cardiology, Baylor Scott & White Medical Center, College Station, Texas, USA

**Keywords:** Effective orifice area, Kansas City Cardiomyopathy Questionnaire, Patient–prosthesis mismatch, NYHA class, Quality of life, Transcatheter aortic valve implantation

## Abstract

**Background:**

While patient–prosthesis mismatch (PPM) after transcatheter aortic valve implantation (TAVI) has not been associated with increased mortality, its impact on quality of life (QoL) remains unclear.

**Methods:**

We retrospectively analyzed 3013 patients undergoing TAVI (2012-2022) within a large health care system. Patients were stratified by effective orifice area indexed to body surface area (EOAi) into no (EOAi >0.85 cm^2^/m^2^), moderate (EOAi >0.65 cm^2^/m^2^ or ≤0.85 cm^2^/m^2^), or severe (EOAi ≤0.65 cm^2^/m^2^) PPM, with lower cutoffs for obese patients (body mass index ≥30 kg/m^2^).

**Results:**

The median age was 80.0 (73.0; 86.0) years, and 55.6% were female with a median Society of Thoracic Surgery risk score of 4.70% (2.66; 7.64). Overall, TAVI led to significant improvements in New York Heart Association and Kansas City Cardiomyopathy Questionnaire scores at 30 days and 1 year. Severe predicted and severe measured PPm was not associated with inferior QoL outcomes improvement (all *p* > 0.05).

**Conclusion:**

In this large cohort, TAVI yielded substantial and durable QoL gains, regardless of PPM severity or valve type. These findings suggest that moderate or severe PPM does not diminish functional recovery and should not be a primary determinant in valve selection or procedural strategy at 1 year.

## Highlights

• Large real-world TAVR cohort with structured quality-of-life follow-up.
• Quality of life improved at 30 days and 1 year after TAVR.
• Improvements were consistent regardless of prosthesis–patient mismatch.
• Findings held across mismatch presence, type, and severity categories.
• Suggests valve mismatch has limited impact on post-TAVR quality of life.

## Graphical abstract

**Figure:**
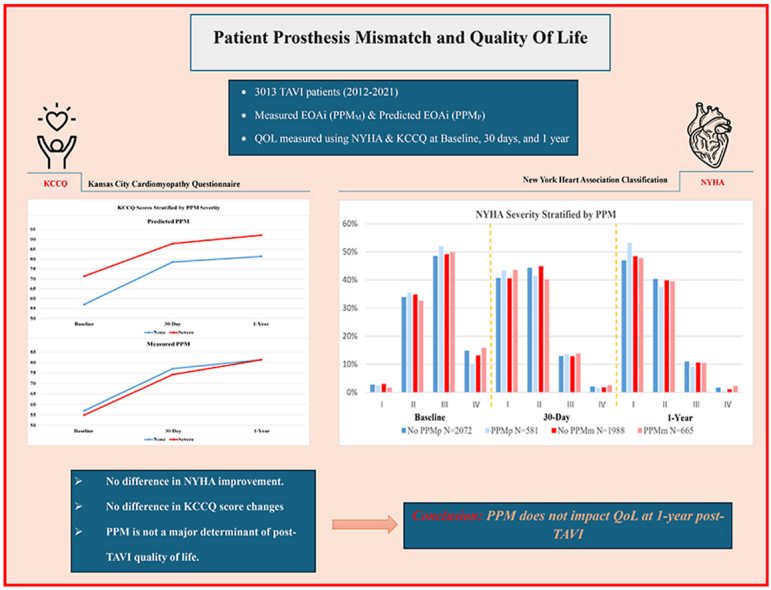

## Introduction

Patient–prosthesis mismatch (PPM) occurs when the effective orifice area (EOA) of a normally functioning prosthetic valve is too small relative to the patient’s body surface area (BSA), leading to increased transprosthetic pressure gradients.^1^^,^^2^ While PPM following surgical aortic valve replacement has been associated with adverse clinical outcomes,^3^^,^^4^ the prognostic significance of PPM following transcatheter aortic valve implantation (TAVI) remains debated. Some studies have demonstrated no association between PPM and mortality after TAVI,^5^, ^6^, ^7^ whereas others have noted a possible impact in select patient subsets.^7^^,^^8^ Methodological heterogeneity, particularly in defining PPM via predicted versus measured EOA, may contribute to these inconsistent findings.^9^^,^^10^ Recent analyses, however, suggest that PPM following TAVI does not increase the risk of mortality, even in patients with small annuli.^11^^,^^12^

Despite this generally reassuring survival evidence, relying solely on mortality metrics may overlook important dimensions of patient well-being. Quality of life (QoL), encompassing symptom burden and functional capacity, is increasingly recognized as a key metric of success following aortic valve interventions. Accordingly, we evaluated the short- and long-term QoL impact of both predicted (PPM_P_) and measured (PPM_M_) PPM in patients undergoing de novo TAVI. By evaluating changes in the Kansas City Cardiomyopathy Questionnaire (KCCQ) and New York Heart Association (NYHA) classification, we aimed to determine whether PPM compromises functional recovery or overall well-being post-TAVI. Understanding this relationship may facilitate a more refined patient selection process, prosthesis choice, and post-TAVI management strategies to enhance patient-centered care.

## Methods

This retrospective cohort study included consecutive patients who underwent TAVI for severe native aortic stenosis between January 2012 and December 2022 at the Baylor Scott & White Health-Care System. All patients received commercially available balloon-expanding valves (BEVs) (Sapien, Sapien XT, Sapien 3 [Edwards Lifesciences]) or self-expanding valves (SEVs) (CoreValve, Evolut R, Evolut Pro [Medtronic]). Consistent with prior protocols, patients undergoing valve-in-valve TAVI were excluded, as were those lacking postoperative echocardiographic assessments.^11^ Clinical, procedural, and echocardiographic variables were retrospectively extracted from the electronic medical record (Epic Systems). QoL following TAVI was assessed utilizing NYHA class and overall KCCQ scores collected preoperatively and again at 30 days and 1 year.

Echocardiographic parameters were obtained from transthoracic echocardiography performed at each site and interpreted by experienced echocardiographers. A central core laboratory and formal reproducibility analyses were not employed, reflecting real-world post-TAVR practice. EOA was derived via the continuity equation and indexed to BSA (EOAi).^2^^,^^13^ Measured EOAi was obtained directly via transthoracic echocardiography–based calculations, while predicted EOAi was estimated using published normal reference values for each prosthesis size and type, then normalized by patient BSA.^14^ PPM was defined according to the Valve Academic Research Consortium -3 consensus criteria. Severe PPM was defined as EOAi ≤0.65 cm^2^/m^2^ (≤0.55 cm^2^/m^2^ for patients with body mass index ≥30 kg/m^2^). Moderate PPM was defined as EOAi >0.65 but ≤0.85 cm^2^/m^2^ (>0.55 but ≤0.70 cm^2^/m^2^ in obese patients). PPM was considered absent when EOAi exceeded these cutoffs. Both measured EOAi (PPM_M_) and predicted EOAi (PPM_P_) were assessed.

The primary endpoint was the change in QoL from baseline to 30 days and 1 year, based on NYHA functional class and patient-reported KCCQ scores. Analyses of NYHA transitions were stratified by baseline (pre-TAVR) NYHA classification.

Secondary endpoints included changes in QoL measures according to the presence of moderate or severe PPM (both PPM_P_ and PPM_M_) at 30 days and 1 year, as well as the impact of PPM on QoL outcomes within each valve type group over the same time periods.

Six comparison groups were analyzed to evaluate QoL outcomes, including NYHA class and changes in KCCQ scores: no PPM_P_ vs. any PPM_P_, no PPM_P_ vs. moderate PPM_P_, no PPM_P_ vs. severe PPM_P_, no PPM_M_ vs. any PPM_M_, no PPM_M_ vs. moderate PPM_M_, and no PPM_M_ vs. severe PPM_M_. The Kolmogorov-Smirnov test was employed to assess the normality of continuous variables. All analyses were conducted using the original, nonimputed data set to maintain transparency and avoid bias introduced by imputation. To compare the change of KCCQ between groups, analysis of covariance was performed, adjusting for covariates including age, gender, high surgical risk (Society of Thoracic Surgeons score ≥8), low-flow status (stroke volume index <35 on postoperative echocardiography), and use of a nontransfemoral approach. Postoperative NYHA class was compared using chi-square tests, with stratification by baseline NYHA class to account for differences in baseline (preoperative) class. To account for multiple comparisons, the Bonferroni correction was applied to 6 predefined comparisons for each QoL measure in analysis with either the full study cohort or subsets of patients. The corrected alpha level was set at 0.0083 (0.05/6). *p* values below this threshold were considered statistically significant. All analyses were conducted in R (version 4.4.0; R Foundation for Statistical Computing).

## Results

### Patient Population and Baseline Characteristics

A total of 3013 patients underwent TAVI for severe aortic stenosis during the study period. The median age was 80.0 years (interquartile range [IQR]: 73.0–86.0), and 55.6% (N = 1676) were female (Table 1). Comorbidities were common, including hypertension (89.3%), diabetes (40.6%), and coronary artery disease (18.8%), resulting in a median Society of Thoracic Surgeons Procedural Risk of Mortality score of 4.70% (2.66–7.64). At baseline, the median aortic valve gradient was 43.0 mmHg (38.0–50.0) with a median valve area of 0.70 cm^2^ (0.60–0.80). Most patients (63.1%) presented with NYHA class III or IV symptoms with a median KCCQ score of 57.1 (41.4–72.9).

**Table 1:** Demographic, baseline echocardiographic, and quality of life metrics

Baseline clinical and demographics	All patients (N = 3013)
Age, y	80.0 [73.0–86.0]
Sex	
Male	1337 (44.4)
Female	1676 (55.6)
BMI, kg/m^2^	27.9 [24.3–32.4]
BMI ≥30 kg/m^2^	1115 (37.0)
BSA, m^2^	1.96 [1.77–2.15]
STS-PROM score	4.70% [2.66–7.64]
Hypertension	2688 (89.3)
Diabetes	1222 (40.6)
CAD	567 (18.8)
AF or flutter	1036 (35.0)
Pacemaker	436 (14.5)
Prior cardiac surgery	656 (21.9)
Creatinine, mg/dL	1.15 [0.94–1.45]
PAD	651 (21.6)
Baseline echocardiography	
Median aortic valve gradient, mmHg	43.0 [38.0–50.0]
Median aortic valve area, cm^2^	0.70 [0.60–0.80]
Aortic regurgitation (≥moderate)	392 (13.0)
Baseline NYHA	
Class I	71 (2.7)
Class II	908 (34.2)
Class III	1308 (49.3)
Class IV	366 (13.8)
Baseline KCCQ	57.1 [41.4–72.9]

Values are presented as median [interquartile range] for continuous variables and as count (percentage) for categorical variables.

Baseline KCCQ and NYHA class were captured before the procedure.

Abbreviations: AF, atrial fibrillation; BMI, body mass index; BSA, body surface area; CAD, coronary artery disease; KCCQ, Kansas City Cardiomyopathy Questionnaire; NYHA, New York Heart Association; PAD, peripheral artery disease; STS-PROM, Society of Thoracic Surgeons Predicted Risk of Mortality.

### Procedural Characteristics and Postoperative Echocardiography

A transfemoral approach was used in 95.3% of the cohort, and 6.1% of procedures were classified as urgent or emergent (Table 2). BEVs were implanted in 1967 patients (65.3%), whereas 686 (22.8%) received SEVs. The median implanted valve size was 26.0 mm (23.0–29.0). Postprocedure echocardiography demonstrated a median left ventricular ejection fraction of 60.0% (54.0–65.0) with a prosthetic mean gradient of 9.0 mmHg (6.0–12.0). A residual mean gradient of ≥20 mmHg was observed in 3.4% of patients. The prevalence of overall PPM_P_ was 21.8%, with 0.8% categorized as severe. The prevalence of PPM_M_ was slightly higher at 25.3%, with 6.3% of patients qualifying as severe.

**Table 2:** Procedural and postprocedural echocardiographic characteristics and PPM

Procedural characteristics	All patients (N = 3013)
Urgent/emergent procedure	93 (6.14%)
Transfemoral approach	2871 (95.3%)
Valve type	
BEV	1967 (65.3)
SEV	686 (22.8)
Valve size, mm	26.0 [23.0–29.0]
Postoperative echocardiography	
LVEF %	60.0 [54.0–65.0]
Prosthetic mean gradient, mmHg	9.00 [6.00–12.00]
High residual gradient (mean gradient ≥20 mmHg)	102 (3.4)
Measured EOA, cm^2^/m^2^	1.90 [1.55–2.30]
Measured EOAi, cm^2^/m^2^	0.96 [0.79–1.17]
Moderate or severe aortic regurgitation	92 (3.1)
SVI, ml/m^2^	36.5 [29.5–45.2]
SVI ≤35 ml/m^2^	947 (45.0)
Predicted PPM	
Predicted EOAi, cm2/m2	0.88 [0.79–0.98]
Overall predicted PPM	657 (21.8)
Predicted PPM severity	
None	2356 (78.2)
Moderate	633 (21.0)
Severe	24 (0.8)
Measured PPM	
Measured EOAi, cm2/m2	0.96 [0.79–1.17]
Overall measured PPM	761 (25.3)
Measured PPM severity	
None	2252 (74.7)
Moderate	572 (19.0)
Severe	189 (6.3)

Values are presented as median [interquartile range] for continuous variables and as count (percentage) for categorical variables.

Abbreviations: BEV, balloon-expandable valve; EOA, effective orifice area; EOAi, indexed effective orifice area; LVEF, left ventricular ejection fraction; PPM, prosthesis-patient mismatch; SEV, self-expanding valve; SVI, stroke volume index.

### Postprocedure QoL and NYHA Class by Measured and Predicted PPM

Overall, TAVI led to significant improvements in both NYHA class and KCCQ scores. At 30 days, only 15.0% of patients were NYHA III/IV (down from 63.1% at baseline), and the median KCCQ rose to 77.1 (62.9–87.1), representing a median improvement of 14.3 points (2.85–28.6). At 1 year, 11.9% remained in NYHA III/IV, and the median KCCQ had further increased to 81.4 (67.1–90.0).

When stratified by PPM_P_, among patients in NYHA I–II at baseline, 93.8% without PPM_P_ versus 94.2% with overall PPM_P_ remained in class I–II at 30 days (*p* = 0.991); results were similar at 1 year (93.7 vs. 95.8%; *p* = 0.500). Among those in NYHA III–IV at baseline, functional improvement was frequent, with 80.4% without PPM_P_ versus 80.4% with overall PPM_P_ improving to I–II at 30 days (*p* = 1.000) and 84.3 versus 87.3% at 1 year (*p* = 0.359). Median improvement from baseline to 30 days was 12.9 (IQR: 1.43–28.6) without PPM_P_ vs. 14.3 (2.86–27.5) overall (*p* = 0.575); at 1 year, 14.3 (2.86–28.6) vs. 15.7 (2.86–30.0), respectively (*p* = 0.977).

### No vs. Severe/No vs. Moderate

**Figure 1:**
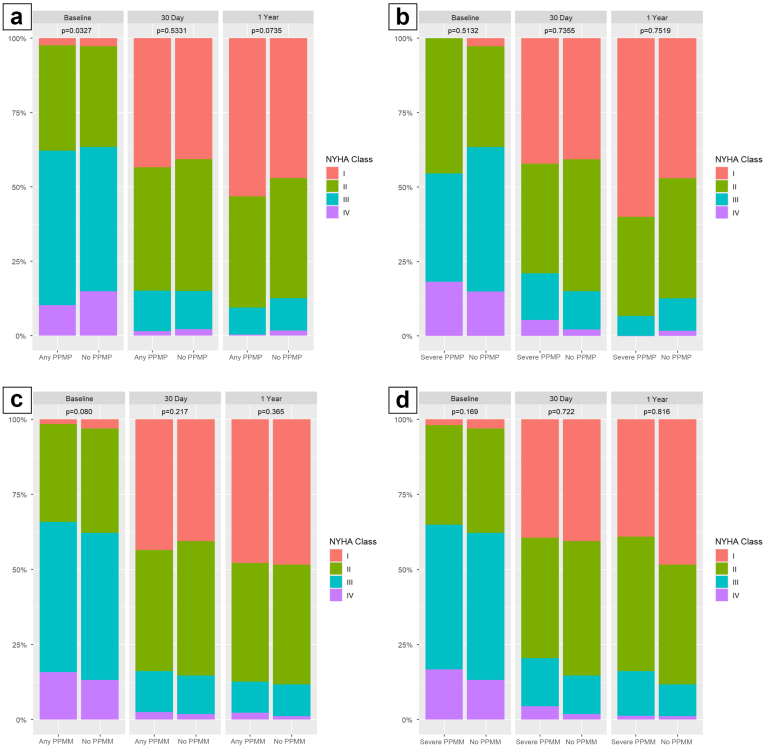
NYHA classification at baseline, 30 days, and 1 year stratified by PPM_P_ (a and b) and PPM_M_ (c and d). Abbreviations: NYHA, New York Heart Association; PPM_P_, predicted prosthesis–patient mismatch; PPM_M_, measured prosthesis–patient mismatch.

At 30 days, 93.9% of baseline I–II patients with moderate PPM_P_ (*p* = 1.000) and 100% with severe PPM_P_ (*p* = 1.000) remained in class I–II, compared with 93.8% without PPM_P_. For baseline III–IV, 80.8% with moderate (*p* = 0.930) and 66.7% with severe PPM_P_ (*p* = 0.392) improved to I–II vs. 80.4% without PPM_P_. At 1 year, 95.6% with moderate (*p* = 0.589) and 100% with severe PPM_P_ (*p* = 1.000) of baseline I–II patients remained in class I–II, compared with 93.7% without PPM_P_; among baseline III–IV, 87.3% with moderate (*p* = 0.371) and 87.5% with severe PPM_P_ (*p* = 1.000) improved vs. 84.3% without PPM_P_. At 30 days, median improvements were 14.3 (2.86–27.5) for moderate (*p* = 0.498) and 15.0 (5.35–28.9) for severe PPM_P_ (*p* = 0.658) vs. 12.9 without PPM_P_. At 1 year, improvements were 15.7 (2.86–30.0) (*p* = 0.864) and 18.6 (4.65–26.4) (*p* = 0.333) vs. 14.3 without PPM_P_ (Table 3, Figure 1a and b).

**Table 3:** Comparisons in NYHA class and KCCQ scores stratified by predicted PPM

	No PPM_P_	Overall PPM_P_	*p* value	No PPM_P_	Moderate PPM_P_	*p* value	No PPM_P_	Severe PPM_P_	*p* value
Baseline NYHA I or II									
NYHA 30 d (N = 784)									
I or II	574 (93.8%)	162 (94.2%)	0.991	574 (93.8%)	153 (93.9%)	1	574 (93.8%)	9 (100%)	1
III or IV	38 (6.21%)	10 (5.81%)		38 (6.21%)	10 (6.13%)		38 (6.21%)	0 (0.00%)	
NYHA 1 y (N = 579)									
I or II	430 (93.7%)	115 (95.8%)	0.5	430 (93.7%)	108 (95.6%)	0.589	430 (93.7%)	7 (100%)	1
III or IV	29 (6.32%)	5 (4.17%)		29 (6.32%)	5 (4.42%)		29 (6.32%)	0 (0.00%)	
Baseline NYHA III or IV									
NYHA 30 d (N = 1319)									
I or II	843 (80.4%)	217 (80.4%)	1	843 (80.4%)	211 (80.8%)	0.93	843 (80.4%)	6 (66.7%)	0.392
III or IV	206 (19.6%)	53 (19.6%)		206 (19.6%)	50 (19.2%)		206 (19.6%)	3 (33.3%)	
NYHA 1 y (N = 921)									
I or II	617 (84.3%)	165 (87.3%)	0.359	617 (84.3%)	158 (87.3%)	0.371	617 (84.3%)	7 (87.5%)	1
III or IV	115 (15.7%)	24 (12.7%)		115 (15.7%)	23 (12.7%)		115 (15.7%)	1 (12.5%)	
	No PPM_P_ (N = 1596)	Overall PPM_P_ (N = 460)	*p* value	No PPM_P_ (N = 1596)	Moderate PPM_P_ (N = 440)	*p* value	No PPM_P_ (N = 1596)	Severe PPM_P_ (N = 20)	*p* value
KCCQ change	12.9	14.3	0.575	12.9	14.3	0.498	12.9	15	0.658
Baseline-30 d	[1.43–28.6]	[2.86–27.5]		[1.43–28.6]	[2.86–27.5]		[1.43–28.6]	[5.35–28.9]	
KCCQ change	14.3	15.7	0.977	14.3	15.7	0.864	14.3	18.6	0.333
Baseline-1 y	[2.86–28.6]	[2.86–30.0]		[2.86–28.6]	[2.86–30.0]		[2.86–28.6]	[4.65–26.4]	

Values are presented as n (%) or median [interquartile range]. *p* values compare patients without predicted patient–prosthesis mismatch (PPM_P_) to those with overall, moderate, or severe PPM_P_.

Baseline KCCQ and NYHA class were captured before the procedure.

Abbreviations: KCCQ, Kansas City Cardiomyopathy Questionnaire; NYHA, New York Heart Association; PPM, prosthesis-patient mismatch; PPM_P_, predicted patient–prosthesis mismatch.

When stratified by PPM_M_, among baseline I–II patients, 93.4% without PPM_M_ vs. 95.3% with overall PPM_M_ remained in I–II at 30 days (*p* = 0.435); at 1 year, 94.6 vs. 92.5% (*p* = 0.494). Among baseline III–IV, 82.9% without PPM_M_ vs. 78.2% with overall PPM_M_ improved at 30 days (*p* = 0.285), and 86.2 vs. 85.2% at 1 year (*p* = 0.980). At 30 days, median improvement was 12.9 (IQR: 2.85–27.1) without PPM_M_ vs. 15.7 (2.86–28.6) overall (*p* = 0.655); at 1 year, 14.3 (2.86–28.6) vs. 17.1 (2.85–30.0) (*p* = 0.630).

### No vs. Severe/No vs. Moderate

At 30 days, 95.9% of baseline I–II patients with moderate PPM_M_ (*p* = 0.362) and 93.6% with severe PPM_M_ (*p* = 1.000) remained in I–II, compared with 93.4% without PPM_M_. Among baseline III–IV, 80.6% with moderate (*p* = 0.938) and 70.7% with severe PPM_M_ (unadjusted *p* = 0.034; not significant after Bonferroni correction) improved vs. 82.9% without PPM_M_. At 1 year, results remained similar: 94.1% with moderate (*p* = 1.000) and 87.5% with severe PPM_M_ (*p* = 0.109) of baseline I–II patients remained in class I–II vs. 94.6% without PPM_M_; among baseline III–IV, 86.6% with moderate (*p* = 0.634) and 80.0% with severe PPM_M_ (*p* = 0.480) improved vs. 86.2% without PPM_M_. At 30 days, median improvements were 15.7 (2.86–28.6) for moderate (*p* = 0.851) and 15.7 (4.28–28.6) for severe PPM_M_ (*p* = 0.399) vs. 12.9 without PPM_M_. At 1 year, improvements were 15.7 (2.86–30.0) (*p* = 0.361) and 18.6 (5.00–33.2) (*p* = 0.482) vs. 14.3 without PPM_M_ (Table 4).

**Table 4:** Comparisons in NYHA class and KCCQ scores stratified by measured PPM

	No PPM_M_	Overall PPM_M_	*p* value	No PPM_M_	Moderate PPM_M_	*p* value	No PPM_M_	Severe PPM_M_	*p* value
Baseline NYHA I or II									
NYHA 30 d (N = 784)									
I or II	553 (93.4%)	183 (95.3%)	0.435	553 (93.4%)	139 (95.9%)	0.362	553 (93.4%)	44 (93.6%)	1
III or IV	39 (6.59%)	9 (4.69%)		39 (6.59%)	6 (4.14%)		39 (6.59%)	3 (6.38%)	
NYHA 1 y (N = 579)									
I or II	421 (94.6%)	124 (92.5%)	0.494	421 (94.6%)	96 (94.1%)	1	421 (94.6%)	28 (87.5%)	0.109
III or IV	24 (5.39%)	10 (7.46%)		24 (5.39%)	6 (5.88%)		24 (5.39%)	4 (12.5%)	
Baseline NYHA III or IV									
NYHA 30 d (N = 1319)									
I or II	798 (81.1%)	262 (78.2%)	0.285	798 (81.1%)	204 (80.6%)	0.938	798 (81.1%)	58 (70.7%)	0.034∗
III or IV	186 (18.9%)	73 (21.8%)		186 (18.9%)	49 (19.4%)		186 (18.9%)	24 (29.3%)	
NYHA 1 y (N = 921)									
I or II	581 (84.8%)	201 (85.2%)	0.98	581 (84.8%)	161 (86.6%)	0.634	581 (84.8%)	40 (80.0%)	0.48
III or IV	104 (15.2%)	35 (14.8%)		104 (15.2%)	25 (13.4%)		104 (15.2%)	10 (20.0%)	
	No PPM_M_ (N = 1557)	Overall PPM_M_ (N = 499)	*p* value	No PPM_M_ (N = 1557)	Moderate PPM_M_ (N = 378)	*p* value	No PPM_M_ (N = 1557)	Severe PPM_M_ (N = 121)	*p* value
KCCQ change	12.9	15.7	0.655	12.9	15.7	0.851	12.9	15.7	0.399
Baseline-30 d	[2.85–27.1]	[2.86–28.6]		[1.43–28.6]	[2.86–28.6]		[2.85–27.1]	[4.28–28.6]	
KCCQ change	14.3	17.1	0.63	14.3	15.7	0.361	14.3	18.6	0.482
Baseline-1 y	[2.86–28.6]	[2.85–30.0]		[2.86–28.6]	[2.86–30.0]		[2.86–28.6]	[5.00–33.2]	

Values are presented as n (%) or median [interquartile range]. *p* values compare patients without measured patient–prosthesis mismatch (PPM_M_) to those with overall, moderate, or severe PPM_M_.

Abbreviations: KCCQ, Kansas City Cardiomyopathy Questionnaire; NYHA, New York Heart Association; PPM, prosthesis-patient mismatch; PPM_M_, measured patient–prosthesis mismatch.

∗ Did not reach statistical significance after Bonferroni correction.

Overall, these analyses demonstrate that transitions in functional status and improvements in QoL scores were comparable across patients with no, moderate, or severe PPM, whether assessed by predicted or measured methods, with no evidence of differential impact at either 30 days or 1 year.

### Postprocedure QoL and NYHA Class by Valve Type

#### SEV Recipients

##### No PPM_M_ vs. Overall PPM_M_

Among baseline NYHA I–II patients, 91.0% without PPM_M_ vs. 94.9% with overall PPM_M_ remained in class I–II at 30 days (*p* = 0.654), and 92.6 vs. 93.8% at 1 year> (*p* = 1.000) (Supplementary Tables 1 and 2). Among baseline III–IV patients, 82.9% without PPM_M_ vs. 76.5% with overall PPM_M_ improved to class I–II at 30 days (*p* = 0.275), with similar proportions at 1 year (86.2 vs. 85.7%; *p* = 1.000). KCCQ improvements were also comparable: median gains were 12.9 (IQR: 1.43–28.6) vs. 18.6 (5.72–31.5) at 30 days (*p* = 0.326), and 14.3 (1.79–30.0) vs. 17.9 (1.43–28.6) at 1 year (*p* = 0.529).

##### No PPM_M_ vs. Moderate/Severe PPM_M_

At 30 days, 92.6% with moderate (*p* = 1.000) and 100% with severe PPM_M_ (*p* = 0.591) remained in I–II vs. 91.0% without PPM_M_. At 1 year, 92.0% with moderate (*p* = 1.000) and 100% with severe PPM_M_ (*p* = 1.000) vs. 92.6% without PPM_M_ remained in I–II. For baseline NYHA III–IV patients, 76.0% with moderate (*p* = 0.319) and 77.8% with severe PPM_M_ (*p* = 0.807) improved to I–II at 30 days, vs. 82.9% without PPM_M_; by 1 year, 86.1% (moderate, *p* = 1.000) and 84.6% (severe, *p* = 1.000) vs. 86.2% without PPM_M_ were in I–II. KCCQ scores showed similar patterns: median improvements were 18.6 (5.72–30.4) (moderate, *p* = 0.777) and 17.1 (5.71–33.6) (severe, *p* = 0.124) vs. 12.9 (1.43–28.6) without PPM_M_ at 30 days, and 20.0 (2.85–30.0) (moderate, *p* = 0.429) and 12.9 (1.43–28.6) (severe, *p* = 0.975) vs. 14.3 (1.79–30.0) at 1 year.

##### No PPM_P_ vs. Overall PPM_P_

Among baseline I–II patients, 91.2% without PPM_P_ vs. 100% with overall PPM_P_ remained in I–II at 30 days (*p* = 0.515), and 92.3 vs. 100% at 1 year (*p* = 0.782). Among baseline III–IV patients, 81.7% without PPM_P_ vs. 84.6% with overall PPM_P_ improved to I–II at 30 days (*p* = 0.910), and 85.5 vs. 95.0% at 1 year (*p* = 0.392). Median KCCQ improvements were 14.3 (1.43–29.6) vs. 19.3 (3.22–34.3) at 30 days (*p* = 0.594) and 14.3 (1.43–30.0) vs. 14.3 (4.29–24.3) at 1 year (*p* = 0.623).

##### No PPM_P_ vs. Moderate/Severe PPM_P_

At 30 days, 100% with moderate (*p* = 0.897) and 100% with severe PPM_P_ (*p* = 0.897) remained in I–II vs. 91.2% without PPM_P_. At 1 year, 100% with moderate (*p* = 1.000) and 100% with severe PPM_P_ (*p* = 1.000) vs. 92.3% without PPM_P_ remained in I–II. Among baseline III–IV patients, 94.1% with moderate (*p* = 0.323) and 66.7% with severe PPM_P_ (*p* = 0.478) improved to I–II at 30 days, vs. 81.7% without PPM_P_; by 1 year, 100% with moderate (*p* = 0.318) and 87.5% with severe PPM_P_ (*p* = 1.000) were in I–II, compared with 85.5% without PPM_P_. KCCQ improvements were also similar: 21.4 (6.07–32.5) in moderate (*p* = 0.612) and 12.9 (–0.36–34.6) in severe PPM_P_ (*p* = 0.769) vs. 14.3 (1.43–29.6) at 30 days; and 14.3 (4.28–20.0] (moderate, *p* = 0.896) and 11.4 (4.65–27.5) (severe, *p* = 0.304) vs. 14.3 (1.43–30.0] at 1 year.

#### BEV Recipients

##### No PPM_M_ vs. Overall PPM_M_

Among baseline I–II patients, 94.2% without PPM_M_ vs. 95.4% with overall PPM_M_ remained in I–II at 30 days (*p* = 0.707), and 95.4 vs. 92.2% at 1 year (*p* = 0.317) (Supplementary Tables 3 and 4). Among baseline III–IV patients, 80.1% without PPM_M_ vs. 78.7% with overall PPM_M_ improved to I–II at 30 days (*p* = 0.680), and 84.1 vs. 85.0% at 1 year (*p* = 0.852). Median KCCQ improvement was 12.9 (2.85–27.1) vs. 14.3 (2.86–28.6) at 30 days (*p* = 0.856), and 14.3 (2.86–28.6) vs. 17.1 (2.86–30.0) at 1 year (*p* = 0.932).

##### No PPM_M_ vs. Moderate/Severe PPM_M_

At 30 days, 96.6% with moderate (*p* = 0.415) and 91.4% with severe PPM_M_ (*p* = 0.771) remained in I–II vs. 94.2% without PPM_M_. At 1 year, 94.8% with moderate (*p* = 1.000) and 84.0% with severe PPM_M_ (*p* = 0.050) vs. 95.4% without PPM_M_ remained in I–II. Among baseline III–IV patients, 81.8% with moderate (*p* = 0.678) and 68.8% with severe PPM_M_ improved to I–II at 30 days, vs. 80.1% without PPM_M_. The signal for severe PPM_M_ reached nominal significance (*p* = 0.048) but was not significant after Bonferroni correction. At 1 year, 86.7% with moderate (*p* = 0.525) and 78.4% with severe PPM_M_ (*p* = 0.506) were in I–II, vs. 84.1% without PPM_M_. Median KCCQ improvement was 14.3 (2.86–28.6) (moderate, *p* = 0.843) and 14.3 (3.24–28.2) (severe, *p* = 0.877) vs. 12.9 (2.85–27.1) at 30 days; and 15.7 (1.60–28.6) (moderate, *p* = 0.571) and 20.0 (7.17–35.0) (severe, *p* = 0.308) vs. 14.3 (2.86–28.6) at 1 year.

##### No PPM_P_ vs. Overall PPM_P_

Among baseline I–II patients, 94.8% without PPM_P_ vs. 93.7% with overall PPM_P_ remained in I–II at 30 days (*p* = 0.742), and 94.3 vs. 95.5% at 1 year (*p* = 0.830). Among baseline III–IV patients, 79.6% without PPM_P_ vs. 79.9% with overall PPM_P_ improved to I–II at 30 days (*p* = 0.993), and 83.6 vs. 86.4% at 1 year (*p* = 0.464). KCCQ gains were also comparable: 12.9 (2.85–27.2) vs. 14.3 (3.22–27.1) at 30 days (*p* = 0.525), and 14.3 (2.86–28.6) vs. 15.7 (2.86–30.0) at 1 year (*p* = 0.822).

##### No PPM_P_ vs. Moderate/Severe PPM_P_

At 30 days, 93.6% with moderate (*p* = 0.716) and 100% with severe PPM_P_ (*p* = 1.000) remained in I–II, vs. 94.8% without PPM_P_. At 1 year, 95.3% with moderate (*p* = 0.875) and 100% with severe PPM_P_ (*p* = 1.000) remained in I–II, vs. 94.3% without PPM_P_. For baseline III–IV patients, 79.9% with moderate improved to I–II at 30 days (*p* = 0.993), compared with 79.6% without PPM_P_; no severe cases were observed. At 1 year, 86.4% with moderate PPM_P_ (*p* = 0.464) improved to I–II vs. 83.6% without PPM_P_. Median KCCQ improvements were 14.3 (2.86–27.1] in moderate (*p* = 0.525) and 16.4 (13.6–17.9) in severe PPM_P_ (*p* = 0.920) vs. 12.9 (2.85–27.2) at 30 days; and 15.7 (2.86–30.0) in moderate (*p* = 0.815) and 21.4 (16.4–22.9) in severe PPM_P_ (*p* = 0.969) vs. 14.3 (2.86–28.6) at 1 year.

Overall, across both SEV and BEV recipients, neither measured nor predicted PPM, whether overall, moderate, or severe, was associated with significant differences in NYHA class transitions or KCCQ improvement at 30 days or 1 year.

## Discussion

This large retrospective study highlights several key findings. First, TAVI is associated with significant early (30-day) and sustained (1-year) improvement in patient-reported QoL, as measured by NYHA functional class and KCCQ scores. Second, the presence of PPM, whether predicted or measured, did not negatively impact QoL improvements. Third, within each valve type (BEV and SEV), PPM, whether measured or predicted and across all severities, was not associated with differential changes in QoL. Patients experienced comparable improvements in KCCQ scores from baseline to 30 days and 1 year, irrespective of PPM status.

Following TAVI, patients experienced substantial and sustained improvements in both NYHA class and KCCQ scores. At baseline, 63.1% of patients were in NYHA class III/IV and had a median KCCQ score of 57.1. By 30 days, only 15.0% remained in NYHA III/IV, and the median KCCQ score rose to 77.1. At 1 year, NYHA class III/IV decreased further to 11.9%, and the KCCQ score increased to 81.4, yielding a median 14.3-point improvement from baseline. These data underscore the marked and durable symptomatic relief afforded by TAVI, echoing the findings of prior investigations that similarly demonstrate early functional and QoL benefits following TAVI.^15^, ^16^, ^17^, ^18^, ^19^ For instance, Hiltrop et al. reported clinically meaningful QoL gains up to 2 years in a high-risk cohort,^16^ and more recent data in low-risk patients also indicate early KCCQ improvements that persist over time.^18^

Consistent with other contemporary TAVI studies, our data revealed no detrimental impact of moderate or severe PPM (whether predicted or measured) on NYHA class transition or KCCQ improvements at 30 days or 1 year.^5^^,^^9^^,^^20^ Specifically, among patients with severe PPM_P_, 33.3% remained in NYHA class III/IV at 30 days, decreasing to 12.5% at 1 year, reflecting progressive functional improvement comparable to the non-PPM group. Similarly, KCCQ scores improved comparably between groups, with a median increase of 15.0 points at 30 days and 18.6 points at 1 year for severe PPM_P_, versus 12.9 and 14.3 points in the non-PPM group, respectively. Findings were similar in the PPM_M_ cohort, where severe PPM_M_ showed no significant impact on QoL improvements, with KCCQ gains at 1 year comparable to those in patients without PPM_M_.

These results were similar to those reported by Herrmann et al., who analyzed over 60,000 TAVI recipients from the transcatheter valve therapy registry and found that while severe PPM was associated with slightly lower KCCQ improvement at 30 days (27.4 ± 26.8 vs. 31.1 ± 27.4 for no PPM), at 1 year the difference was no longer present.^9^ Similarly, a meta-analysis conducted by Sá et al. reported no significant impact between PPM and QoL at 1 year post-TAVI.^21^ Additional studies examining the impact of higher post-TAVI gradients, which can be influenced by PPM, have likewise shown minimal effect on QoL.^22^^,^^23^ Although PPM may carry hemodynamic implications post-TAVI, our data and others’ support the conclusion that it does not adversely affect short- or mid-term QoL outcomes.

Patients undergoing TAVI with SEVs and BEVs represent distinct clinical populations and were therefore analyzed separately rather than directly compared. Within each valve type, transitions in NYHA functional class at 30 days and 1 year, as well as changes in KCCQ scores, demonstrated consistent improvement from baseline irrespective of PPM status. Importantly, neither measured nor predicted PPM, whether moderate or severe, was associated with adverse impact on functional or QoL outcomes at 1 year. These findings corroborate prior research showing no significant long-term QoL differences between BEVs and SEVs.^17^^,^^18^^,^^24^, ^25^, ^26^ Overall, these findings highlight that although BEV and SEV recipients represent distinct populations with differing baseline functional status, both valve types are associated with comparable long-term improvements in QoL following TAVI, even in the presence of measured or predicted PPM.

Overall, these results suggest that TAVI provides substantial QoL benefits across a broad spectrum of patients, regardless of baseline functional status, valve type, or PPM classification. The data suggest that moderate or severe PPM need not drive aggressive procedural modifications specifically to mitigate PPM risk in most cases, especially since QoL improvements remain robust. Additionally, as SEVs and BEVs demonstrated comparable QoL outcomes, prosthesis selection should be patient-specific, considering valve longevity, the need for future valve-in-valve TAVI, as well as future cardiac catheterizations. Further research is warranted to evaluate longer-term QoL differences beyond the 1-year mark and its relationship to structural valve deterioration over time.^25^

### Limitations

This study’s retrospective, observational design introduces potential residual confounders that may not be fully controlled. Moreover, we used NYHA functional class and KCCQ as our primary QoL metrics; though validated, these may not capture the entire spectrum of patient-reported health status (e.g., mental and social well-being) and represent subjective measures of overall well-being. The study period spanned a decade (2012–2022), during which multiple generations and models of transcatheter valves were used. Device heterogeneity may have influenced outcomes and represents an inherent limitation of the study. Our follow-up was limited to 1 year, thus preventing evaluation of longer-term QoL trajectories and potential late influences of PPM on structural valve deterioration. Finally, although the large, multicenter cohort improves generalizability, variations in procedural techniques, operator expertise, and iterative device enhancements over time may also affect outcomes.

## Conclusion

TAVI resulted in substantial and sustained QoL improvements across all patient groups, with rapid symptomatic relief and durable functional recovery at 1 year. Importantly, these benefits persisted even in the setting of moderate or severe PPM and across different valve types. These findings underscore the broad applicability of TAVI in contemporary practice and highlight that, for most patients, PPM should not be a primary deterrent in prosthesis selection or procedural planning.

### Impact on Daily Practice

This study underscores that moderate or severe PPM, whether measured or predicted, does not significantly compromise short- or mid-term QoL improvements after TAVI. Even patients with smaller annuli or reduced left ventricular ejection fraction derive comparable symptom relief and functional recovery. Consequently, clinicians may be less inclined to employ aggressive procedural measures solely to prevent PPM, as overall outcomes remain robust. In practical terms, prosthesis selection can be tailored more flexibly, focusing on patient anatomy, valve durability, and anticipated future interventions.

## Ethical Statement

This retrospective study was approved by the institutional review board and Ethics Committee at Baylor Scott & White Health (IRB# 014-209). The research was conducted in accordance with the principles of the Declaration of Helsinki and institutional ethical standards. Owing to the retrospective nature of the analysis, the requirement for individual patient informed consent was waived by the ethics committee.

## Funding

This study was supported by a grant from the Cardiovascular Research Review Committee (CVRRC) at 10.13039/100020125Baylor Scott & White Research Institute, with additional philanthropic support from Satish and Yasmin Gupta, the 10.13039/100020125Baylor Scott & White Dallas Foundation, and the Roberts Foundation.

## Disclosure Statement

K. Al-Azizi is a proctor and consultant for Edwards Lifesciences; is a consultant and advisory board member for Medtronic; is a consultant for Boston Scientific; and is a speaker for Philips. M. Szerlip is a proctor, speaker, and consultant for Edwards Lifesciences; is an advisory board member, consultant, and proctor for Abbott Vascular; is a member of Medtronic’s steering committee; and is a speaker for Boston Scientific. R. Stoler is a proctor and advisory board member for Medtronic, Boston Scientific, and Edwards; and is an advisory board member for Biotronik. M.J. Mack is a trial co-principal investigator for Abbott and Edwards Lifesciences, and a trial study chair for Medtronic (all roles uncompensated). S. Potluri is an advisory board member, proctor, and speaker for Medtronic, Boston Scientific, Abbott, and Cordis; and is a proctor and speaker for Edwards, Terumo, and AstraZeneca. J.C. Contreras is a proctor for Edwards Lifesciences. The other authors had no conflicts to declare.

## References

[1] Pibarot P., Clavel M.A. (2018). Prosthesis-patient mismatch after transcatheter aortic valve replacement: it is neither rare nor benign. J Am Coll Cardiol.

[2] Lancellotti P., Pibarot P., Chambers J. (2016). Recommendations for the imaging assessment of prosthetic heart valves: a report from the European association of cardiovascular imaging endorsed by the Chinese society of echocardiography, the inter-American society of echocardiography, and the Brazilian department of cardiovascular imaging. Eur Heart J - Cardiovasc Imaging.

[3] Fallon J.M., DeSimone J.P., Brennan J.M. (2018). The incidence and consequence of prosthesis-patient mismatch after surgical aortic valve replacement. Ann Thorac Surg.

[4] Head S.J., Mokhles M.M., Osnabrugge R.L.J. (2012). The impact of prosthesis–patient mismatch on long-term survival after aortic valve replacement: a systematic review and meta-analysis of 34 observational studies comprising 27 186 patients with 133 141 patient-years. Eur Heart J.

[5] Thyregod H.G.H., Steinbrüchel D.A., Ihlemann N. (2016). No clinical effect of prosthesis–patient mismatch after transcatheter versus surgical aortic valve replacement in intermediate- and low-risk patients with severe aortic valve stenosis at mid-term follow-up: an analysis from the NOTION trial. Eur J Cardio-Thoracic Surg.

[6] Pibarot P., Weissman N.J., Stewart W.J. (2014). Incidence and sequelae of prosthesis-patient mismatch in transcatheter versus surgical valve replacement in high-risk patients with severe aortic stenosis: a PARTNER trial cohort--a analysis. J Am Coll Cardiol.

[7] Levesque T., Eltchaninoff H., Chabannes R. (2024). Impact of prosthesis-patient mismatch after transcatheter aortic valve replacement. Can J Cardiol.

[8] Schofer N., Deuschl F., Rübsamen N. (2019). Prosthesis-patient mismatch after transcatheter aortic valve implantation: prevalence and prognostic impact with respect to baseline left ventricular function. EuroIntervention.

[9] Herrmann H.C., Daneshvar S.A., Fonarow G.C. (2018). Prosthesis–patient mismatch in patients undergoing transcatheter aortic valve replacement: from the STS/ACC TVT registry. J Am Coll Cardiol.

[10] Tang G.H.L., Sengupta A., Alexis S.L. (2021). Outcomes of prosthesis-patient mismatch following supra-annular transcatheter aortic valve replacement: from the STS/ACC TVT registry. JACC Cardiovasc Interv.

[11] Al-Azizi K., Moubarak G., Mohiuddin A. (2024). Five-year outcomes of measured and predicted prosthesis-patient mismatch following transcatheter aortic valve implantation. Am J Cardiol.

[12] Tchétché D., Mehran R., Blackman D.J. (2024). Transcatheter aortic valve implantation by valve type in women with small annuli: results from the SMART randomized clinical trial. JAMA Cardiol.

[13] Zoghbi W.A., Chambers J.B., Dumesnil J.G. (2009). Recommendations for evaluation of prosthetic valves with echocardiography and doppler ultrasound: a report from the American society of echocardiography’s guidelines and standards committee and the task force on prosthetic valves, developed in conjunction with the American college of cardiology cardiovascular imaging committee, Cardiac imaging committee of the American heart association, the European association of echocardiography, a registered branch of the European society of cardiology, the Japanese society of echocardiography and the Canadian society of echocardiography, endorsed by the American college of cardiology foundation, American heart association, European association of echocardiography, a registered branch of the European society of cardiology, the Japanese society of echocardiography, and Canadian society of echocardiography. J Am Soc Echocardiogr.

[14] Hahn R.T., Leipsic J., Douglas P.S. (2019). Comprehensive echocardiographic assessment of normal transcatheter valve function. JACC Cardiovasc Imaging.

[15] Dahiya G., Kyvernitakis A., Joshi A.A. (2021). Impact of transcatheter aortic valve replacement on left ventricular hypertrophy, diastolic dysfunction and quality of life in patients with preserved left ventricular function. Int J Cardiovasc Imaging.

[16] Hiltrop N., Belmans A., Claes M. (2016). Functional performance and quality of life in high-risk comorbid patients undergoing transcatheter aortic valve implantation for symptomatic aortic valve stenosis. Eur Heart J Qual Care Clin Outcomes.

[17] Nuche J., Abbas A.E., Serra V. (2023). Balloon- vs self-expanding transcatheter valves for failed small surgical aortic bioprostheses. JACC Cardiovasc Interv.

[18] Modine T., Tchétché D., Van Mieghem N.M. (2024). Three-year outcomes following TAVR in younger (<75 years) low-surgical-risk severe aortic stenosis patients. Circ Cardiovasc Interv.

[19] Arnold S.V., Spertus J.A., Vemulapalli S. (2015). Association of patient-reported health status with long-term mortality after transcatheter aortic valve replacement: report from the STS/ACC TVT registry. Circ Cardiovasc Interv.

[20] Ahmad D., Sá M.P., Makani A. (2024). Transcatheter aortic valve replacement in patients with small aortic annuli: a propensity-matched analysis of gender-based outcomes. Am J Cardiol.

[21] Sá M.P., Jacquemyn X., Van den Eynde J. (2023). Impact of prosthesis-patient mismatch after transcatheter aortic valve replacement: meta-analysis of Kaplan-Meier–Derived individual patient data. JACC Cardiovasc Imaging.

[22] Alperi A., Robichaud M., Panagides V. (2022). Impact of residual transvalvular gradient on clinical outcomes following valve-in-valve transcatheter aortic valve replacement. Int J Cardiol.

[23] Kherallah R.Y., Suffredini J.M., Rahman F. (2024). Impact of elevated gradients after transcatheter aortic valve implantation for degenerated surgical aortic valve bioprostheses. Circ Cardiovasc Interv.

[24] Abdel-Wahab M., Landt M., Neumann F.-J. (2020). 5-Year outcomes after TAVR with balloon-expandable versus self-expanding valves: results from the CHOICE randomized clinical trial. JACC Cardiovasc Interv.

[25] Herrmann H.C., Mehran R., Blackman D.J. (2024). Self-expanding or balloon-expandable TAVR in patients with a small aortic annulus. N Engl J Med.

[26] Okuno T., Khan F., Asami M. (2019). Prosthesis-patient mismatch following transcatheter aortic valve replacement with supra-annular and intra-annular prostheses. JACC Cardiovasc Interv.

