# Supplementary material for: Impact of Measured and Predicted Patient–Prosthesis Mismatch on Quality of Life Following Transcatheter Aortic Valve Implantation
Source: Struct Heart. 2025 Nov 10;10(1):100759. doi: 10.1016/j.shj.2025.100759 (PMC12796100; doi:10.1016/j.shj.2025.100759)
Supplement: Supplementary File [file mmc1.docx]

## **Supplementary Table 1. SEV Patients, NYHA Class and KCCQ by BMI-adjusted predicted PPM (None vs Moderate)**

|  | **None** | **Moderate** | **p-value** | **N** |
| --- | --- | --- | --- | --- |
|  | ***N=637*** | ***N=31*** |  |  |
| Baseline NYHA: |  |  | 0.295 | 668 |
| I | 12 (1.88%) | 1 (3.23%) |  |  |
| II | 178 (27.9%) | 8 (25.8%) |  |  |
| III | 313 (49.1%) | 19 (61.3%) |  |  |
| IV | 134 (21.0%) | 3 (9.68%) |  |  |
| NYHA 30 Days Post-op: |  |  | 0.585 | 594 |
| I | 183 (32.1%) | 8 (33.3%) |  |  |
| II | 299 (52.5%) | 15 (62.5%) |  |  |
| III | 79 (13.9%) | 1 (4.17%) |  |  |
| IV | 9 (1.58%) | 0 (0.00%) |  |  |
| NYHA 1 Year Post-op: |  |  | 0.058 | 448 |
| I | 169 (39.3%) | 13 (72.2%) |  |  |
| II | 207 (48.1%) | 5 (27.8%) |  |  |
| III | 45 (10.5%) | 0 (0.00%) |  |  |
| IV | 9 (2.09%) | 0 (0.00%) |  |  |
| Baseline NYHA: |  |  | 1.000 | 668 |
| 1. I or II | 190 (29.8%) | 9 (29.0%) |  |  |
| 2. III or IV | 447 (70.2%) | 22 (71.0%) |  |  |
| NYHA 30 Days Post-op: |  |  | 0.236 | 594 |
| 1. I or II | 482 (84.6%) | 23 (95.8%) |  |  |
| 2. III or IV | 88 (15.4%) | 1 (4.17%) |  |  |
| NYHA 1 Year Post-op: |  |  | 0.147 | 448 |
| 1. I or II | 376 (87.4%) | 18 (100%) |  |  |
| 2. III or IV | 54 (12.6%) | 0 (0.00%) |  |  |
| Baseline KCCQ | 54.3 [38.6;70.0] | 54.3 [36.8;66.1] | 0.733 | 563 |
| KCCQ 30 Days Post-op | 75.7 [60.0;87.1] | 77.9 [64.6;85.7] | 0.713 | 526 |
| KCCQ 1 Year Post-op | 80.0 [64.3;88.6] | 78.6 [60.0;87.1] | 0.769 | 403 |
| KCCQ Change: Baseline to 30 Days Post-op | 14.3 [1.43;29.6] | 21.4 [6.07;32.5] | 0.329 | 476 |
| KCCQ Change: Baseline to 1 Year Post-op | 14.3 [1.43;30.0] | 14.3 [4.28;20.0] | 0.761 | 352 |

## **Supplementary Table 2. SEV Patients, NYHA Class and KCCQ by BMI-adjusted measured PPM (None vs Moderate)**

|  | **None** | **Moderate** | **p-value** | **N** |
| --- | --- | --- | --- | --- |
|  | ***N=566*** | ***N=86*** |  |  |
| Baseline NYHA: |  |  | 0.331 | 652 |
| I | 12 (2.12%) | 0 (0.00%) |  |  |
| II | 154 (27.2%) | 28 (32.6%) |  |  |
| III | 288 (50.9%) | 38 (44.2%) |  |  |
| IV | 112 (19.8%) | 20 (23.3%) |  |  |
| NYHA 30 Days Post-op: |  |  | 0.220 | 579 |
| I | 154 (30.7%) | 29 (37.7%) |  |  |
| II | 273 (54.4%) | 34 (44.2%) |  |  |
| III | 67 (13.3%) | 14 (18.2%) |  |  |
| IV | 8 (1.59%) | 0 (0.00%) |  |  |
| NYHA 1 Year Post-op: |  |  | 0.189 | 439 |
| I | 151 (40.1%) | 27 (43.5%) |  |  |
| II | 180 (47.7%) | 28 (45.2%) |  |  |
| III | 41 (10.9%) | 4 (6.45%) |  |  |
| IV | 5 (1.33%) | 3 (4.84%) |  |  |
| Baseline NYHA: |  |  | 0.629 | 652 |
| 1. I or II | 166 (29.3%) | 28 (32.6%) |  |  |
| 2. III or IV | 400 (70.7%) | 58 (67.4%) |  |  |
| NYHA 30 Days Post-op: |  |  | 0.572 | 579 |
| 1. I or II | 427 (85.1%) | 63 (81.8%) |  |  |
| 2. III or IV | 75 (14.9%) | 14 (18.2%) |  |  |
| NYHA 1 Year Post-op: |  |  | 1.000 | 439 |
| 1. I or II | 331 (87.8%) | 55 (88.7%) |  |  |
| 2. III or IV | 46 (12.2%) | 7 (11.3%) |  |  |
| Baseline KCCQ | 54.3 [38.6;70.0] | 52.9 [37.1;65.7] | 0.726 | 548 |
| KCCQ 30 Days Post-op | 75.7 [60.0;87.1] | 75.7 [58.6;86.1] | 0.850 | 514 |
| KCCQ 1 Year Post-op | 78.6 [63.9;88.6] | 79.3 [65.7;88.6] | 0.818 | 390 |
| KCCQ Change: Baseline to 30 Days Post-op | 12.9 [1.43;28.6] | 18.6 [5.72;30.4] | 0.198 | 465 |
| KCCQ Change: Baseline to 1 Year Post-op | 14.3 [1.79;30.0] | 20.0 [2.85;30.0] | 0.319 | 341 |

## **Supplementary Table 3. SEV Patients, NYHA Class and KCCQ by BMI-adjusted predicted PPM (None vs Severe)**

|  | **None** | **Severe** | **p-value** | **N** |
| --- | --- | --- | --- | --- |
|  | ***N=637*** | ***N=18*** |  |  |
| Baseline NYHA: |  |  | 0.676 | 655 |
| I | 12 (1.88%) | 0 (0.00%) |  |  |
| II | 178 (27.9%) | 7 (38.9%) |  |  |
| III | 313 (49.1%) | 7 (38.9%) |  |  |
| IV | 134 (21.0%) | 4 (22.2%) |  |  |
| NYHA 30 Days Post-op: |  |  | 0.309 | 587 |
| I | 183 (32.1%) | 6 (35.3%) |  |  |
| II | 299 (52.5%) | 7 (41.2%) |  |  |
| III | 79 (13.9%) | 3 (17.6%) |  |  |
| IV | 9 (1.58%) | 1 (5.88%) |  |  |
| NYHA 1 Year Post-op: |  |  | 0.870 | 442 |
| I | 169 (39.3%) | 6 (50.0%) |  |  |
| II | 207 (48.1%) | 5 (41.7%) |  |  |
| III | 45 (10.5%) | 1 (8.33%) |  |  |
| IV | 9 (2.09%) | 0 (0.00%) |  |  |
| Baseline NYHA: |  |  | 0.571 | 655 |
| 1. I or II | 190 (29.8%) | 7 (38.9%) |  |  |
| 2. III or IV | 447 (70.2%) | 11 (61.1%) |  |  |
| NYHA 30 Days Post-op: |  |  | 0.322 | 587 |
| 1. I or II | 482 (84.6%) | 13 (76.5%) |  |  |
| 2. III or IV | 88 (15.4%) | 4 (23.5%) |  |  |
| NYHA 1 Year Post-op: |  |  | 1.000 | 442 |
| 1. I or II | 376 (87.4%) | 11 (91.7%) |  |  |
| 2. III or IV | 54 (12.6%) | 1 (8.33%) |  |  |
| Baseline KCCQ | 54.3 [38.6;70.0] | 55.7 [32.9;64.3] | 0.681 | 556 |
| KCCQ 30 Days Post-op | 75.7 [60.0;87.1] | 70.7 [61.4;82.5] | 0.746 | 518 |
| KCCQ 1 Year Post-op | 80.0 [64.3;88.6] | 82.1 [67.9;92.5] | 0.490 | 394 |
| KCCQ Change: Baseline to 30 Days Post-op | 14.3 [1.43;29.6] | 12.9 [-0.36;34.6] | 0.945 | 470 |
| KCCQ Change: Baseline to 1 Year Post-op | 14.3 [1.43;30.0] | 11.4 [4.65;27.5] | 0.989 | 347 |

## **Supplementary Table 4. SEV Patients, NYHA Class and KCCQ by BMI-adjusted measured PPM (None vs Severe)**

|  | **None** | **Severe** | **p-value** | **N** |
| --- | --- | --- | --- | --- |
|  | ***N=566*** | ***N=34*** |  |  |
| Baseline NYHA: |  |  | 0.380 | 600 |
| I | 12 (2.12%) | 1 (2.94%) |  |  |
| II | 154 (27.2%) | 11 (32.4%) |  |  |
| III | 288 (50.9%) | 13 (38.2%) |  |  |
| IV | 112 (19.8%) | 9 (26.5%) |  |  |
| NYHA 30 Days Post-op: |  |  | 0.076 | 534 |
| I | 154 (30.7%) | 14 (43.8%) |  |  |
| II | 273 (54.4%) | 14 (43.8%) |  |  |
| III | 67 (13.3%) | 2 (6.25%) |  |  |
| IV | 8 (1.59%) | 2 (6.25%) |  |  |
| NYHA 1 Year Post-op: |  |  | 0.373 | 398 |
| I | 151 (40.1%) | 10 (47.6%) |  |  |
| II | 180 (47.7%) | 9 (42.9%) |  |  |
| III | 41 (10.9%) | 1 (4.76%) |  |  |
| IV | 5 (1.33%) | 1 (4.76%) |  |  |
| Baseline NYHA: |  |  | 0.585 | 600 |
| 1. I or II | 166 (29.3%) | 12 (35.3%) |  |  |
| 2. III or IV | 400 (70.7%) | 22 (64.7%) |  |  |
| NYHA 30 Days Post-op: |  |  | 1.000 | 534 |
| 1. I or II | 427 (85.1%) | 28 (87.5%) |  |  |
| 2. III or IV | 75 (14.9%) | 4 (12.5%) |  |  |
| NYHA 1 Year Post-op: |  |  | 1.000 | 398 |
| 1. I or II | 331 (87.8%) | 19 (90.5%) |  |  |
| 2. III or IV | 46 (12.2%) | 2 (9.52%) |  |  |
| Baseline KCCQ | 54.3 [38.6;70.0] | 55.0 [35.7;70.4] | 0.832 | 505 |
| KCCQ 30 Days Post-op | 75.7 [60.0;87.1] | 85.7 [70.4;87.5] | 0.067 | 470 |
| KCCQ 1 Year Post-op | 78.6 [63.9;88.6] | 82.9 [78.6;90.0] | 0.136 | 355 |
| KCCQ Change: Baseline to 30 Days Post-op | 12.9 [1.43;28.6] | 17.1 [5.71;33.6] | 0.263 | 428 |
| KCCQ Change: Baseline to 1 Year Post-op | 14.3 [1.79;30.0] | 12.9 [1.43;28.6] | 0.887 | 311 |

## **Supplementary Table 5. BEV Patients, NYHA Class and KCCQ by BMI-adjusted measured PPM (None vs Severe)**

|  | **None** | **Severe** | **p-value** | **N** |
| --- | --- | --- | --- | --- |
|  | ***N=1422*** | ***N=128*** |  |  |
| Baseline NYHA: |  |  | 0.379 | 1550 |
| I | 48 (3.38%) | 2 (1.56%) |  |  |
| II | 537 (37.8%) | 43 (33.6%) |  |  |
| III | 688 (48.4%) | 65 (50.8%) |  |  |
| IV | 149 (10.5%) | 18 (14.1%) |  |  |
| NYHA 30 Days Post-op: |  |  | 0.098 | 1301 |
| I | 534 (44.6%) | 40 (38.1%) |  |  |
| II | 489 (40.9%) | 41 (39.0%) |  |  |
| III | 151 (12.6%) | 20 (19.0%) |  |  |
| IV | 22 (1.84%) | 4 (3.81%) |  |  |
| NYHA 1 Year Post-op: |  |  | 0.044 | 896 |
| I | 434 (52.3%) | 24 (36.4%) |  |  |
| II | 301 (36.3%) | 30 (45.5%) |  |  |
| III | 87 (10.5%) | 12 (18.2%) |  |  |
| IV | 8 (0.96%) | 0 (0.00%) |  |  |
| Baseline NYHA: |  |  | 0.220 | 1550 |
| 1. I or II | 585 (41.1%) | 45 (35.2%) |  |  |
| 2. III or IV | 837 (58.9%) | 83 (64.8%) |  |  |
| NYHA 30 Days Post-op: |  |  | 0.031 | 1301 |
| 1. I or II | 1023 (85.5%) | 81 (77.1%) |  |  |
| 2. III or IV | 173 (14.5%) | 24 (22.9%) |  |  |
| NYHA 1 Year Post-op: |  |  | 0.154 | 896 |
| 1. I or II | 735 (88.6%) | 54 (81.8%) |  |  |
| 2. III or IV | 95 (11.4%) | 12 (18.2%) |  |  |
| Baseline KCCQ | 58.6 [44.3;74.3] | 55.0 [39.6;68.6] | 0.025 | 1532 |
| KCCQ 30 Days Post-op | 78.6 [62.9;88.6] | 71.4 [60.4;85.4] | 0.022 | 1337 |
| KCCQ 1 Year Post-op | 81.4 [68.6;90.0] | 81.4 [65.0;87.1] | 0.405 | 1017 |
| KCCQ Change: Baseline to 30 Days Post-op | 12.9 [2.85;27.1] | 14.3 [3.24;28.2] | 0.616 | 1250 |
| KCCQ Change: Baseline to 1 Year Post-op | 14.3 [2.86;28.6] | 20.0 [7.17;35.0] | 0.041 | 943 |

## **Supplementary Table 6. BEV Patients, NYHA Class and KCCQ by BMI-adjusted measured PPM (None vs Moderate)**

|  | **None** | **Moderate** | **p-value** | **N** |
| --- | --- | --- | --- | --- |
|  | ***N=1422*** | ***N=417*** |  |  |
| Baseline NYHA: |  |  | 0.030 | 1839 |
| I | 48 (3.38%) | 8 (1.92%) |  |  |
| II | 537 (37.8%) | 135 (32.4%) |  |  |
| III | 688 (48.4%) | 216 (51.8%) |  |  |
| IV | 149 (10.5%) | 58 (13.9%) |  |  |
| NYHA 30 Days Post-op: |  |  | 0.845 | 1544 |
| I | 534 (44.6%) | 162 (46.6%) |  |  |
| II | 489 (40.9%) | 137 (39.4%) |  |  |
| III | 151 (12.6%) | 41 (11.8%) |  |  |
| IV | 22 (1.84%) | 8 (2.30%) |  |  |
| NYHA 1 Year Post-op: |  |  | 0.582 | 1076 |
| I | 434 (52.3%) | 128 (52.0%) |  |  |
| II | 301 (36.3%) | 89 (36.2%) |  |  |
| III | 87 (10.5%) | 24 (9.76%) |  |  |
| IV | 8 (0.96%) | 5 (2.03%) |  |  |
| Baseline NYHA: |  |  | 0.014 | 1839 |
| 1. I or II | 585 (41.1%) | 143 (34.3%) |  |  |
| 2. III or IV | 837 (58.9%) | 274 (65.7%) |  |  |
| NYHA 30 Days Post-op: |  |  | 0.926 | 1544 |
| 1. I or II | 1023 (85.5%) | 299 (85.9%) |  |  |
| 2. III or IV | 173 (14.5%) | 49 (14.1%) |  |  |
| NYHA 1 Year Post-op: |  |  | 0.973 | 1076 |
| 1. I or II | 735 (88.6%) | 217 (88.2%) |  |  |
| 2. III or IV | 95 (11.4%) | 29 (11.8%) |  |  |
| Baseline KCCQ | 58.6 [44.3;74.3] | 55.7 [41.4;72.9] | 0.143 | 1797 |
| KCCQ 30 Days Post-op | 78.6 [62.9;88.6] | 78.6 [64.3;87.5] | 0.962 | 1579 |
| KCCQ 1 Year Post-op | 81.4 [68.6;90.0] | 81.4 [65.7;90.0] | 0.734 | 1223 |
| KCCQ Change: Baseline to 30 Days Post-op | 12.9 [2.85;27.1] | 14.3 [2.86;28.6] | 0.260 | 1470 |
| KCCQ Change: Baseline to 1 Year Post-op | 14.3 [2.86;28.6] | 15.7 [1.60;28.6] | 0.974 | 1125 |

## **Supplementary Table 7. BEV Patients, NYHA Class and KCCQ by BMI-adjusted predicted PPM (None vs Moderate)**

|  | **None** | **Moderate** | **p-value** | **N** |
| --- | --- | --- | --- | --- |
|  | ***N=1435*** | ***N=528*** |  |  |
| Baseline NYHA: |  |  | 0.328 | 1963 |
| I | 45 (3.14%) | 13 (2.46%) |  |  |
| II | 524 (36.5%) | 188 (35.6%) |  |  |
| III | 693 (48.3%) | 275 (52.1%) |  |  |
| IV | 173 (12.1%) | 52 (9.85%) |  |  |
| NYHA 30 Days Post-op: |  |  | 0.570 | 1647 |
| I | 543 (44.8%) | 191 (44.0%) |  |  |
| II | 491 (40.5%) | 176 (40.6%) |  |  |
| III | 151 (12.4%) | 61 (14.1%) |  |  |
| IV | 28 (2.31%) | 6 (1.38%) |  |  |
| NYHA 1 Year Post-op: |  |  | 0.441 | 1139 |
| I | 429 (51.0%) | 154 (51.7%) |  |  |
| II | 306 (36.4%) | 114 (38.3%) |  |  |
| III | 94 (11.2%) | 29 (9.73%) |  |  |
| IV | 12 (1.43%) | 1 (0.34%) |  |  |
| Baseline NYHA: |  |  | 0.559 | 1963 |
| 1. I or II | 569 (39.7%) | 201 (38.1%) |  |  |
| 2. III or IV | 866 (60.3%) | 327 (61.9%) |  |  |
| NYHA 30 Days Post-op: |  |  | 0.792 | 1647 |
| 1. I or II | 1034 (85.2%) | 367 (84.6%) |  |  |
| 2. III or IV | 179 (14.8%) | 67 (15.4%) |  |  |
| NYHA 1 Year Post-op: |  |  | 0.291 | 1139 |
| 1. I or II | 735 (87.4%) | 268 (89.9%) |  |  |
| 2. III or IV | 106 (12.6%) | 30 (10.1%) |  |  |
| Baseline KCCQ | 57.1 [43.9;72.9] | 58.6 [41.4;72.9] | 0.709 | 1913 |
| KCCQ 30 Days Post-op | 78.6 [62.9;88.6] | 77.1 [63.9;87.1] | 0.985 | 1677 |
| KCCQ 1 Year Post-op | 81.4 [67.1;90.0] | 82.9 [70.0;89.3] | 0.482 | 1286 |
| KCCQ Change: Baseline to 30 Days Post-op | 12.9 [2.85;27.2] | 14.3 [2.86;27.1] | 0.413 | 1560 |
| KCCQ Change: Baseline to 1 Year Post-op | 14.3 [2.86;28.6] | 15.7 [2.86;30.0] | 0.644 | 1184 |

## **Supplementary Table 8. BEV Patients, NYHA Class and KCCQ by BMI-adjusted predicted PPM (None vs Severe)**

|  | **None** | **Severe** | **p-value** | **N** |
| --- | --- | --- | --- | --- |
|  | ***N=1435*** | ***N=4*** |  |  |
| Baseline NYHA: |  |  | 0.525 | 1439 |
| I | 45 (3.14%) | 0 (0.00%) |  |  |
| II | 524 (36.5%) | 3 (75.0%) |  |  |
| III | 693 (48.3%) | 1 (25.0%) |  |  |
| IV | 173 (12.1%) | 0 (0.00%) |  |  |
| NYHA 30 Days Post-op: |  |  | 0.637 | 1215 |
| I | 543 (44.8%) | 2 (100%) |  |  |
| II | 491 (40.5%) | 0 (0.00%) |  |  |
| III | 151 (12.4%) | 0 (0.00%) |  |  |
| IV | 28 (2.31%) | 0 (0.00%) |  |  |
| NYHA 1 Year Post-op: |  |  | 0.513 | 844 |
| I | 429 (51.0%) | 3 (100%) |  |  |
| II | 306 (36.4%) | 0 (0.00%) |  |  |
| III | 94 (11.2%) | 0 (0.00%) |  |  |
| IV | 12 (1.43%) | 0 (0.00%) |  |  |
| Baseline NYHA: |  |  | 0.307 | 1439 |
| 1. I or II | 569 (39.7%) | 3 (75.0%) |  |  |
| 2. III or IV | 866 (60.3%) | 1 (25.0%) |  |  |
| NYHA 30 Days Post-op: |  |  | 1.000 | 1215 |
| 1. I or II | 1034 (85.2%) | 2 (100%) |  |  |
| 2. III or IV | 179 (14.8%) | 0 (0.00%) |  |  |
| NYHA 1 Year Post-op: |  |  | 1.000 | 844 |
| 1. I or II | 735 (87.4%) | 3 (100%) |  |  |
| 2. III or IV | 106 (12.6%) | 0 (0.00%) |  |  |
| Baseline KCCQ | 57.1 [43.9;72.9] | 71.4 [69.6;73.9] | 0.115 | 1400 |
| KCCQ 30 Days Post-op | 78.6 [62.9;88.6] | 87.9 [86.4;88.6] | 0.111 | 1233 |
| KCCQ 1 Year Post-op | 81.4 [67.1;90.0] | 92.1 [89.3;92.9] | 0.032 | 935 |
| KCCQ Change: Baseline to 30 Days Post-op | 12.9 [2.85;27.2] | 16.4 [13.6;17.9] | 0.889 | 1146 |
| KCCQ Change: Baseline to 1 Year Post-op | 14.3 [2.86;28.6] | 21.4 [16.4;22.9] | 0.727 | 855 |

**Supplementary Table 9. QOL of Patients with LVEF <50% , Group by Predicted PPM Level**

|  | **None** | **Moderate** | **Severe** | **p-value** | **N** |
| --- | --- | --- | --- | --- | --- |
|  | ***N=456*** | ***N=82*** | ***N=3*** |  |  |
| Postop LVEF | 36.0 [30.0;43.8] | 39.0 [30.0;45.0] | 32.2 [25.7;38.0] | 0.351 | 541 |
| Baseline NYHA: |  |  |  | 0.840 | 476 |
| I | 13 (3.23%) | 2 (2.78%) | 0 (0.00%) |  |  |
| II | 100 (24.9%) | 22 (30.6%) | 1 (50.0%) |  |  |
| III | 201 (50.0%) | 32 (44.4%) | 1 (50.0%) |  |  |
| IV | 88 (21.9%) | 16 (22.2%) | 0 (0.00%) |  |  |
| NYHA 30 Days Post-op: |  |  |  | 0.026 | 416 |
| I | 139 (39.2%) | 33 (55.0%) | 1 (100%) |  |  |
| II | 153 (43.1%) | 14 (23.3%) | 0 (0.00%) |  |  |
| III | 51 (14.4%) | 12 (20.0%) | 0 (0.00%) |  |  |
| IV | 12 (3.38%) | 1 (1.67%) | 0 (0.00%) |  |  |
| NYHA 1 Year Post-op: |  |  |  | 1.000 | 252 |
| I | 95 (43.6%) | 15 (44.1%) | 0 (.%) |  |  |
| II | 93 (42.7%) | 15 (44.1%) | 0 (.%) |  |  |
| III | 25 (11.5%) | 4 (11.8%) | 0 (.%) |  |  |
| IV | 5 (2.29%) | 0 (0.00%) | 0 (.%) |  |  |
| Baseline NYHA: |  |  |  | 0.380 | 476 |
| 1. I or II | 113 (28.1%) | 24 (33.3%) | 1 (50.0%) |  |  |
| 2. III or IV | 289 (71.9%) | 48 (66.7%) | 1 (50.0%) |  |  |
| NYHA 30 Days Post-op: |  |  |  | 0.569 | 416 |
| 1. I or II | 292 (82.3%) | 47 (78.3%) | 1 (100%) |  |  |
| 2. III or IV | 63 (17.7%) | 13 (21.7%) | 0 (0.00%) |  |  |
| NYHA 1 Year Post-op: |  |  |  | 1.000 | 252 |
| 1. I or II | 188 (86.2%) | 30 (88.2%) | 0 (.%) |  |  |
| 2. III or IV | 30 (13.8%) | 4 (11.8%) | 0 (.%) |  |  |
| Baseline KCCQ | 50.0 [37.1;67.1] | 50.0 [31.4;67.1] | 45.7 [36.4;55.0] | 0.496 | 435 |
| KCCQ 30 Days Post-op | 77.1 [61.4;87.1] | 76.4 [55.0;88.6] | 80.0 [80.0;80.0] | 0.911 | 398 |
| KCCQ 1 Year Post-op | 81.4 [67.1;90.0] | 77.1 [69.3;90.0] | 92.9 [92.9;92.9] | 0.302 | 274 |
| KCCQ Change: Baseline to 30 Days Post-op | 19.4 [4.29;32.1] | 17.2 [7.86;32.9] | 15.7 [15.7;15.7] | 0.958 | 363 |
| KCCQ Change: Baseline to 1 Year Post-op | 19.4 [5.72;34.3] | 17.1 [4.29;38.6] | 28.6 [28.6;28.6] | 0.857 | 249 |

**Supplementary Table 10. QOL of Patients with LVEF ≥50%, Group by Predicted PPM Level**

|  | **None** | **Moderate** | **Severe** | **p-value** | **N** |
| --- | --- | --- | --- | --- | --- |
|  | ***N=1887*** | ***N=548*** | ***N=21*** |  |  |
| Postop LVEF | 63.0 [60.0;65.0] | 65.0 [60.0;69.0] | 65.0 [60.0;70.0] | <0.001 | 2456 |
| Baseline NYHA: |  |  |  | . | 2164 |
| I | 43 (2.59%) | 11 (2.27%) | 0 (0.00%) |  |  |
| II | 595 (35.8%) | 173 (35.7%) | 9 (45.0%) |  |  |
| III | 803 (48.4%) | 261 (53.9%) | 7 (35.0%) |  |  |
| IV | 219 (13.2%) | 39 (8.06%) | 4 (20.0%) |  |  |
| NYHA 30 Days Post-op: |  |  |  | . | 1832 |
| I | 580 (40.9%) | 164 (41.5%) | 7 (38.9%) |  |  |
| II | 636 (44.8%) | 176 (44.6%) | 7 (38.9%) |  |  |
| III | 178 (12.5%) | 50 (12.7%) | 3 (16.7%) |  |  |
| IV | 25 (1.76%) | 5 (1.27%) | 1 (5.56%) |  |  |
| NYHA 1 Year Post-op: |  |  |  | . | 1343 |
| I | 499 (47.7%) | 151 (53.7%) | 9 (60.0%) |  |  |
| II | 419 (40.0%) | 104 (37.0%) | 5 (33.3%) |  |  |
| III | 113 (10.8%) | 25 (8.90%) | 1 (6.67%) |  |  |
| IV | 16 (1.53%) | 1 (0.36%) | 0 (0.00%) |  |  |
| Baseline NYHA: |  |  |  | 0.819 | 2164 |
| 1. I or II | 638 (38.4%) | 184 (38.0%) | 9 (45.0%) |  |  |
| 2. III or IV | 1022 (61.6%) | 300 (62.0%) | 11 (55.0%) |  |  |
| NYHA 30 Days Post-op: |  |  |  | 0.544 | 1832 |
| 1. I or II | 1216 (85.7%) | 340 (86.1%) | 14 (77.8%) |  |  |
| 2. III or IV | 203 (14.3%) | 55 (13.9%) | 4 (22.2%) |  |  |
| NYHA 1 Year Post-op: |  |  |  | 0.337 | 1343 |
| 1. I or II | 918 (87.7%) | 255 (90.7%) | 14 (93.3%) |  |  |
| 2. III or IV | 129 (12.3%) | 26 (9.25%) | 1 (6.67%) |  |  |
| Baseline KCCQ | 57.1 [42.9;72.9] | 60.0 [42.9;72.9] | 62.9 [39.3;70.7] | 0.934 | 2050 |
| KCCQ 30 Days Post-op | 77.1 [61.4;87.1] | 77.1 [64.3;87.1] | 71.4 [64.3;88.6] | 0.802 | 1816 |
| KCCQ 1 Year Post-op | 81.4 [67.1;90.0] | 82.9 [70.0;88.6] | 88.6 [70.0;92.9] | 0.269 | 1422 |
| KCCQ Change: Baseline to 30 Days Post-op | 12.8 [1.43;27.1] | 14.3 [2.86;27.1] | 14.3 [3.57;30.7] | 0.564 | 1685 |
| KCCQ Change: Baseline to 1 Year Post-op | 12.9 [2.86;28.6] | 15.7 [2.86;30.0] | 15.7 [4.29;24.3] | 0.729 | 1294 |

**Supplementary Table 11. QOL of Moderate Predicted PPM Patients, Group by LVEF (<50% vs ≥50%)**

|  | **LVEF < 50%** | **LVEF > 50%** | **p-value** | **N** |
| --- | --- | --- | --- | --- |
|  | ***N=82*** | ***N=548*** |  |  |
| Postop LVEF | 39.0 [30.0;45.0] | 65.0 [60.0;69.0] | <0.001 | 630 |
| Baseline NYHA: |  |  | 0.005 | 556 |
| I | 2 (2.78%) | 11 (2.27%) |  |  |
| II | 22 (30.6%) | 173 (35.7%) |  |  |
| III | 32 (44.4%) | 261 (53.9%) |  |  |
| IV | 16 (22.2%) | 39 (8.06%) |  |  |
| NYHA 30 Days Post-op: |  |  | 0.011 | 455 |
| I | 33 (55.0%) | 164 (41.5%) |  |  |
| II | 14 (23.3%) | 176 (44.6%) |  |  |
| III | 12 (20.0%) | 50 (12.7%) |  |  |
| IV | 1 (1.67%) | 5 (1.27%) |  |  |
| NYHA 1 Year Post-op: |  |  | 0.575 | 315 |
| I | 15 (44.1%) | 151 (53.7%) |  |  |
| II | 15 (44.1%) | 104 (37.0%) |  |  |
| III | 4 (11.8%) | 25 (8.90%) |  |  |
| IV | 0 (0.00%) | 1 (0.36%) |  |  |
| Baseline NYHA: |  |  | 0.525 | 556 |
| 1. I or II | 24 (33.3%) | 184 (38.0%) |  |  |
| 2. III or IV | 48 (66.7%) | 300 (62.0%) |  |  |
| NYHA 30 Days Post-op: |  |  | 0.170 | 455 |
| 1. I or II | 47 (78.3%) | 340 (86.1%) |  |  |
| 2. III or IV | 13 (21.7%) | 55 (13.9%) |  |  |
| NYHA 1 Year Post-op: |  |  | 0.547 | 315 |
| 1. I or II | 30 (88.2%) | 255 (90.7%) |  |  |
| 2. III or IV | 4 (11.8%) | 26 (9.25%) |  |  |
| Baseline KCCQ | 50.0 [31.4;67.1] | 60.0 [42.9;72.9] | 0.004 | 539 |
| KCCQ 30 Days Post-op | 76.4 [55.0;88.6] | 77.1 [64.3;87.1] | 0.284 | 469 |
| KCCQ 1 Year Post-op | 77.1 [69.3;90.0] | 82.9 [70.0;88.6] | 0.994 | 373 |
| KCCQ Change: Baseline to 30 Days Post-op | 17.2 [7.86;32.9] | 14.3 [2.86;27.1] | 0.198 | 438 |
| KCCQ Change: Baseline to 1 Year Post-op | 17.1 [4.29;38.6] | 15.7 [2.86;30.0] | 0.590 | 347 |

**Supplementary Table 12. QOL of Severe Predicted PPM Patients, Group by LVEF (<50% vs ≥50%)**

|  | **LVEF < 50%** | **LVEF > 50%** | **p-value** | **N** |
| --- | --- | --- | --- | --- |
|  | ***N=3*** | ***N=21*** |  |  |
| Postop LVEF | 32.2 [25.7;38.0] | 65.0 [60.0;70.0] | 0.006 | 24 |
| Baseline NYHA: |  |  | 1.000 | 22 |
| II | 1 (50.0%) | 9 (45.0%) |  |  |
| III | 1 (50.0%) | 7 (35.0%) |  |  |
| IV | 0 (0.00%) | 4 (20.0%) |  |  |
| NYHA 30 Days Post-op: |  |  | 1.000 | 19 |
| I | 1 (100%) | 7 (38.9%) |  |  |
| II | 0 (0.00%) | 7 (38.9%) |  |  |
| III | 0 (0.00%) | 3 (16.7%) |  |  |
| IV | 0 (0.00%) | 1 (5.56%) |  |  |
| NYHA 1 Year Post-op: |  |  | . | 15 |
| I | 0 (0.00%) | 9 (60.0%) |  |  |
| II | 0 (0.00%) | 5 (33.3%) |  |  |
| III | 0 (0.00%) | 1 (6.67%) |  |  |
| Baseline NYHA: |  |  | 1.000 | 22 |
| 1. I or II | 1 (50.0%) | 9 (45.0%) |  |  |
| 2. III or IV | 1 (50.0%) | 11 (55.0%) |  |  |
| NYHA 30 Days Post-op: |  |  | 1.000 | 19 |
| 1. I or II | 1 (100%) | 14 (77.8%) |  |  |
| 2. III or IV | 0 (0.00%) | 4 (22.2%) |  |  |
| NYHA 1 Year Post-op: |  |  | . | 15 |
| 1. I or II | 0 (0.00%) | 14 (93.3%) |  |  |
| 2. III or IV | 0 (0.00%) | 1 (6.67%) |  |  |
| Baseline KCCQ | 45.7 [36.4;55.0] | 62.9 [39.3;70.7] | 0.401 | 21 |
| KCCQ 30 Days Post-op | 80.0 [80.0;80.0] | 71.4 [64.3;88.6] | 0.795 | 20 |
| KCCQ 1 Year Post-op | 92.9 [92.9;92.9] | 88.6 [70.0;92.9] | 0.253 | 14 |
| KCCQ Change: Baseline to 30 Days Post-op | 15.7 [15.7;15.7] | 14.3 [3.57;30.7] | 0.862 | 20 |
| KCCQ Change: Baseline to 1 Year Post-op | 28.6 [28.6;28.6] | 15.7 [4.29;24.3] | 0.263 | 14 |

**Supplementary Table 13. QOL of Patients with LVEF <50%, Group by Measured PPM Level**

|  | **None** | **Moderate** | **Severe** | **p-value** | **N** |
| --- | --- | --- | --- | --- | --- |
|  | ***N=356*** | ***N=133*** | ***N=52*** |  |  |
| Postop LVEF | 37.0 [30.0;44.0] | 35.0 [30.0;42.5] | 37.2 [31.0;44.2] | 0.541 | 541 |
| Baseline NYHA: |  |  |  | . | 476 |
| I | 11 (3.49%) | 2 (1.74%) | 2 (4.35%) |  |  |
| II | 87 (27.6%) | 24 (20.9%) | 12 (26.1%) |  |  |
| III | 161 (51.1%) | 52 (45.2%) | 21 (45.7%) |  |  |
| IV | 56 (17.8%) | 37 (32.2%) | 11 (23.9%) |  |  |
| NYHA 30 Days Post-op: |  |  |  | . | 416 |
| I | 119 (42.5%) | 41 (42.3%) | 13 (33.3%) |  |  |
| II | 109 (38.9%) | 40 (41.2%) | 18 (46.2%) |  |  |
| III | 44 (15.7%) | 12 (12.4%) | 7 (17.9%) |  |  |
| IV | 8 (2.86%) | 4 (4.12%) | 1 (2.56%) |  |  |
| NYHA 1 Year Post-op: |  |  |  | 0.071 | 252 |
| I | 70 (40.7%) | 33 (58.9%) | 7 (29.2%) |  |  |
| II | 74 (43.0%) | 20 (35.7%) | 14 (58.3%) |  |  |
| III | 24 (14.0%) | 2 (3.57%) | 3 (12.5%) |  |  |
| IV | 4 (2.33%) | 1 (1.79%) | 0 (0.00%) |  |  |
| Baseline NYHA: |  |  |  | 0.222 | 476 |
| 1. I or II | 98 (31.1%) | 26 (22.6%) | 14 (30.4%) |  |  |
| 2. III or IV | 217 (68.9%) | 89 (77.4%) | 32 (69.6%) |  |  |
| NYHA 30 Days Post-op: |  |  |  | 0.838 | 416 |
| 1. I or II | 228 (81.4%) | 81 (83.5%) | 31 (79.5%) |  |  |
| 2. III or IV | 52 (18.6%) | 16 (16.5%) | 8 (20.5%) |  |  |
| NYHA 1 Year Post-op: |  |  |  | 0.121 | 252 |
| 1. I or II | 144 (83.7%) | 53 (94.6%) | 21 (87.5%) |  |  |
| 2. III or IV | 28 (16.3%) | 3 (5.36%) | 3 (12.5%) |  |  |
| Baseline KCCQ | 51.4 [38.6;68.6] | 50.0 [33.2;67.1] | 50.0 [35.7;64.3] | 0.386 | 435 |
| KCCQ 30 Days Post-op | 77.1 [61.4;88.6] | 77.1 [57.9;85.7] | 68.6 [51.4;84.3] | 0.203 | 398 |
| KCCQ 1 Year Post-op | 81.4 [66.8;90.0] | 79.3 [67.5;90.0] | 77.1 [62.9;89.3] | 0.966 | 274 |
| KCCQ Change: Baseline to 30 Days Post-op | 18.6 [4.29;31.4] | 20.0 [7.15;35.7] | 15.7 [6.43;32.9] | 0.759 | 363 |
| KCCQ Change: Baseline to 1 Year Post-op | 15.7 [2.86;31.4] | 24.3 [10.0;37.1] | 28.6 [6.43;42.9] | 0.055 | 249 |

**Supplementary Table 14. QOL of Patients with LVEF ≥50%, Group by Measured PPM Level**

|  | **None** | **Moderate** | **Severe** | **p-value** | **N** |
| --- | --- | --- | --- | --- | --- |
|  | ***N=1882*** | ***N=438*** | ***N=136*** |  |  |
| Postop LVEF | 65.0 [60.0;68.0] | 62.5 [60.0;67.0] | 60.0 [55.0;65.0] | 0.001 | 2456 |
| Baseline NYHA: |  |  |  | . | 2164 |
| I | 47 (2.83%) | 6 (1.55%) | 1 (0.86%) |  |  |
| II | 596 (35.9%) | 139 (35.8%) | 42 (36.2%) |  |  |
| III | 812 (48.9%) | 202 (52.1%) | 57 (49.1%) |  |  |
| IV | 205 (12.3%) | 41 (10.6%) | 16 (13.8%) |  |  |
| NYHA 30 Days Post-op: |  |  |  | . | 1832 |
| I | 561 (39.9%) | 149 (45.6%) | 41 (41.8%) |  |  |
| II | 651 (46.3%) | 131 (40.1%) | 37 (37.8%) |  |  |
| III | 173 (12.3%) | 43 (13.1%) | 15 (15.3%) |  |  |
| IV | 22 (1.56%) | 4 (1.22%) | 5 (5.10%) |  |  |
| NYHA 1 Year Post-op: |  |  |  | . | 1343 |
| I | 510 (49.6%) | 122 (48.4%) | 27 (42.9%) |  |  |
| II | 406 (39.5%) | 97 (38.5%) | 25 (39.7%) |  |  |
| III | 103 (10.0%) | 26 (10.3%) | 10 (15.9%) |  |  |
| IV | 9 (0.88%) | 7 (2.78%) | 1 (1.59%) |  |  |
| Baseline NYHA: |  |  |  | 0.844 | 2164 |
| 1. I or II | 643 (38.7%) | 145 (37.4%) | 43 (37.1%) |  |  |
| 2. III or IV | 1017 (61.3%) | 243 (62.6%) | 73 (62.9%) |  |  |
| NYHA 30 Days Post-op: |  |  |  | 0.201 | 1832 |
| 1. I or II | 1212 (86.1%) | 280 (85.6%) | 78 (79.6%) |  |  |
| 2. III or IV | 195 (13.9%) | 47 (14.4%) | 20 (20.4%) |  |  |
| NYHA 1 Year Post-op: |  |  |  | 0.207 | 1343 |
| 1. I or II | 916 (89.1%) | 219 (86.9%) | 52 (82.5%) |  |  |
| 2. III or IV | 112 (10.9%) | 33 (13.1%) | 11 (17.5%) |  |  |
| Baseline KCCQ | 58.6 [43.6;74.3] | 57.1 [41.4;72.9] | 55.7 [38.6;70.0] | 0.523 | 2050 |
| KCCQ 30 Days Post-op | 77.1 [61.8;87.1] | 77.1 [64.3;87.1] | 74.3 [65.7;87.1] | 0.828 | 1816 |
| KCCQ 1 Year Post-op | 81.4 [67.1;90.0] | 81.4 [65.7;90.0] | 82.1 [71.1;87.1] | 0.978 | 1422 |
| KCCQ Change: Baseline to 30 Days Post-op | 12.9 [1.43;27.1] | 14.3 [2.86;28.6] | 15.7 [3.24;27.1] | 0.419 | 1685 |
| KCCQ Change: Baseline to 1 Year Post-op | 13.6 [2.86;28.6] | 14.3 [1.43;27.1] | 17.2 [5.71;28.6] | 0.527 | 1294 |

**Supplementary Table 15. QOL of Moderate Measured PPM Patients, Group by LVEF (<50% vs ≥50%)**

|  | **LVEF < 50%** | **LVEF > 50%** | **p-value** | **N** |
| --- | --- | --- | --- | --- |
|  | ***N=133*** | ***N=438*** |  |  |
| Postop LVEF | 35.0 [30.0;42.5] | 62.5 [60.0;67.0] | <0.001 | 571 |
| Baseline NYHA: |  |  | <0.001 | 503 |
| I | 2 (1.74%) | 6 (1.55%) |  |  |
| II | 24 (20.9%) | 139 (35.8%) |  |  |
| III | 52 (45.2%) | 202 (52.1%) |  |  |
| IV | 37 (32.2%) | 41 (10.6%) |  |  |
| NYHA 30 Days Post-op: |  |  | 0.327 | 424 |
| I | 41 (42.3%) | 149 (45.6%) |  |  |
| II | 40 (41.2%) | 131 (40.1%) |  |  |
| III | 12 (12.4%) | 43 (13.1%) |  |  |
| IV | 4 (4.12%) | 4 (1.22%) |  |  |
| NYHA 1 Year Post-op: |  |  | 0.317 | 308 |
| I | 33 (58.9%) | 122 (48.4%) |  |  |
| II | 20 (35.7%) | 97 (38.5%) |  |  |
| III | 2 (3.57%) | 26 (10.3%) |  |  |
| IV | 1 (1.79%) | 7 (2.78%) |  |  |
| Baseline NYHA: |  |  | 0.005 | 503 |
| 1. I or II | 26 (22.6%) | 145 (37.4%) |  |  |
| 2. III or IV | 89 (77.4%) | 243 (62.6%) |  |  |
| NYHA 30 Days Post-op: |  |  | 0.724 | 424 |
| 1. I or II | 81 (83.5%) | 280 (85.6%) |  |  |
| 2. III or IV | 16 (16.5%) | 47 (14.4%) |  |  |
| NYHA 1 Year Post-op: |  |  | 0.161 | 308 |
| 1. I or II | 53 (94.6%) | 219 (86.9%) |  |  |
| 2. III or IV | 3 (5.36%) | 33 (13.1%) |  |  |
| Baseline KCCQ | 50.0 [33.2;67.1] | 57.1 [41.4;72.9] | 0.003 | 460 |
| KCCQ 30 Days Post-op | 77.1 [57.9;85.7] | 77.1 [64.3;87.1] | 0.387 | 416 |
| KCCQ 1 Year Post-op | 79.3 [67.5;90.0] | 81.4 [65.7;90.0] | 0.865 | 331 |
| KCCQ Change: Baseline to 30 Days Post-op | 20.0 [7.15;35.7] | 14.3 [2.86;28.6] | 0.074 | 378 |
| KCCQ Change: Baseline to 1 Year Post-op | 24.3 [10.0;37.1] | 14.3 [1.43;27.1] | 0.003 | 296 |

**Supplementary Table 16. QOL of Severe Measured PPM Patients, Group by LVEF (<50% vs ≥50%)**

|  | **LVEF < 50%** | **LVEF > 50%** | **p-value** | **N** |
| --- | --- | --- | --- | --- |
|  | ***N=52*** | ***N=136*** |  |  |
| Postop LVEF | 37.2 [31.0;44.2] | 60.0 [55.0;65.0] | <0.001 | 188 |
| Baseline NYHA: |  |  | 0.133 | 162 |
| I | 2 (4.35%) | 1 (0.86%) |  |  |
| II | 12 (26.1%) | 42 (36.2%) |  |  |
| III | 21 (45.7%) | 57 (49.1%) |  |  |
| IV | 11 (23.9%) | 16 (13.8%) |  |  |
| NYHA 30 Days Post-op: |  |  | 0.720 | 137 |
| I | 13 (33.3%) | 41 (41.8%) |  |  |
| II | 18 (46.2%) | 37 (37.8%) |  |  |
| III | 7 (17.9%) | 15 (15.3%) |  |  |
| IV | 1 (2.56%) | 5 (5.10%) |  |  |
| NYHA 1 Year Post-op: |  |  | 0.488 | 87 |
| I | 7 (29.2%) | 27 (42.9%) |  |  |
| II | 14 (58.3%) | 25 (39.7%) |  |  |
| III | 3 (12.5%) | 10 (15.9%) |  |  |
| IV | 0 (0.00%) | 1 (1.59%) |  |  |
| Baseline NYHA: |  |  | 0.539 | 162 |
| 1. I or II | 14 (30.4%) | 43 (37.1%) |  |  |
| 2. III or IV | 32 (69.6%) | 73 (62.9%) |  |  |
| NYHA 30 Days Post-op: |  |  | 1.000 | 137 |
| 1. I or II | 31 (79.5%) | 78 (79.6%) |  |  |
| 2. III or IV | 8 (20.5%) | 20 (20.4%) |  |  |
| NYHA 1 Year Post-op: |  |  | 0.749 | 87 |
| 1. I or II | 21 (87.5%) | 52 (82.5%) |  |  |
| 2. III or IV | 3 (12.5%) | 11 (17.5%) |  |  |
| Baseline KCCQ | 50.0 [35.7;64.3] | 55.7 [38.6;70.0] | 0.088 | 152 |
| KCCQ 30 Days Post-op | 68.6 [51.4;84.3] | 74.3 [65.7;87.1] | 0.077 | 130 |
| KCCQ 1 Year Post-op | 77.1 [62.9;89.3] | 82.1 [71.1;87.1] | 0.742 | 90 |
| KCCQ Change: Baseline to 30 Days Post-op | 15.7 [6.43;32.9] | 15.7 [3.24;27.1] | 0.533 | 121 |
| KCCQ Change: Baseline to 1 Year Post-op | 28.6 [6.43;42.9] | 17.2 [5.71;28.6] | 0.147 | 84 |
